# Home-Based Visual Field Monitoring Devices in Glaucoma Management: A Review of Current Evidence and Barriers to Adoption

**DOI:** 10.7759/cureus.95680

**Published:** 2025-10-29

**Authors:** Hussain Aldaher, Fatima A Alsaadi, Sara Bashier, Amena Ali

**Affiliations:** 1 Pediatrics, Mohammed Bin Rashid University of Medicine and Health Sciences/Dubai Health, Dubai, ARE; 2 Ophthalmology, Mohammed Bin Rashid University of Medicine and Health Sciences/Dubai Health, Dubai, ARE

**Keywords:** digital health technologies, digital health technology, glaucoma management, glaucoma monitoring, home-based monitoring, teleophthalmology, visual field progression, visual field test

## Abstract

Glaucoma, which is a leading cause of irreversible blindness, along with many other conditions, relies on visual field testing, which is crucial for diagnosing, monitoring disease progression, and guiding treatment decisions. The COVID-19 pandemic accelerated the development of home-based visual field monitoring tools within telemedicine. This review summarizes the current evidence on these devices, compares them to standard automated perimetry, and discusses barriers to adoption and future directions for clinical integration.

## Introduction and background

Glaucoma is a progressive optic neuropathy and a leading cause of irreversible blindness worldwide, characterized by the gradual degeneration of the optic nerve and resultant loss of retinal ganglion cells (RGCs), which results in permanent visual field (VF) loss if untreated [1,2]. While elevated intraocular pressure (IOP) is a significant risk factor, glaucoma can occur even with normal IOP, indicating that other factors, such as impaired blood supply and neuroinflammation, contribute to disease progression [1,3-5]. The disease often remains asymptomatic until advanced stages, making early detection challenging and emphasizing the importance of routine eye examinations [1,6]. Structural changes in the optic disc, including cupping and thinning of the neuroretinal rim, are hallmarks of glaucomatous damage [5,7]. Current treatments primarily aim to lower IOP through medications, laser therapy, or surgery, but these interventions only slow progression rather than cure the disease [1,8,9]. Recent research highlights the role of neuroinflammatory processes and glial cell activation in the retina and central visual pathways, suggesting that glaucoma shares mechanisms with other neurodegenerative diseases [5,8]. Despite advances in understanding, the exact triggers and mechanisms underlying RGC loss remain incomplete, and there is ongoing research into neuroprotective and immunomodulatory therapies [2,5,8]. Ultimately, without timely intervention, glaucoma leads to progressive and irreversible visual impairment [1,6,9].

VF monitoring in glaucoma disease is essential for monitoring disease progression and guiding therapeutic interventions. VF progression helps physicians to slow or prevent visual disability [10]. VF tests, such as standard automated perimetry (SAP), are used mainly. The correlation between VF and microstructural changes, such as beta-zone peripapillary atrophy (PPA), is fundamental in understanding the disease progression [11].

There are multiple methods of detecting progression over time, such as measuring VF decaying rates, including mean deviation (MD) rate, VF index (VFI) rate, and glaucoma rate index (GRI). These are the latest and most specific and sensitive methods in detecting fast progressive eyes with decaying VF.

Although in-clinic perimetry is a gold standard for glaucoma monitoring, it has several limitations, such as reported false-positive responses that lead to inaccurate reflection on disease progression [12]. Another limitation is that it often misses the early glaucomatous damage effects [12,13]. Finally, measurement reliability and retest variability are especially variable when they depend on the patient's condition and learning effect.

During the COVID-19 pandemic, the rise of digital health and the need for health telemetry started to develop globally. Home monitoring devices were initially developed as small, portable, and accessible devices to measure parameters such as IOP and VF.

This review aims to explore the current use of home monitoring devices and their potential in glaucoma management. Researchers should also examine the barriers to adoption and implementation in daily practices.

## Review

Methods

This narrative review used a simple approach to critically analyze the current evidence on home-based monitoring devices for glaucoma management. A literature search was conducted in the last 10 years using electronic databases such as PubMed, Scopus, Google Scholar, and Web of Science, using keywords such as “home-based visual field”, “remote perimetry”, “self-administered perimetry”, “glaucoma home monitoring”, and “portable perimetry” to refine the search.

Inclusion criteria were studies involving human participants with glaucoma or at risk of glaucoma; publications describing, validating, or evaluating home-based VF monitoring devices; studies examining accuracy, reliability, usability, patient adherence, or clinical integration; and articles published in English and available in full text.

Exclusion criteria were studies focusing solely on clinic-based VF devices without a home-use component, as well as non-English publications, editorials without primary data, and animal studies.

**Figure 1:**
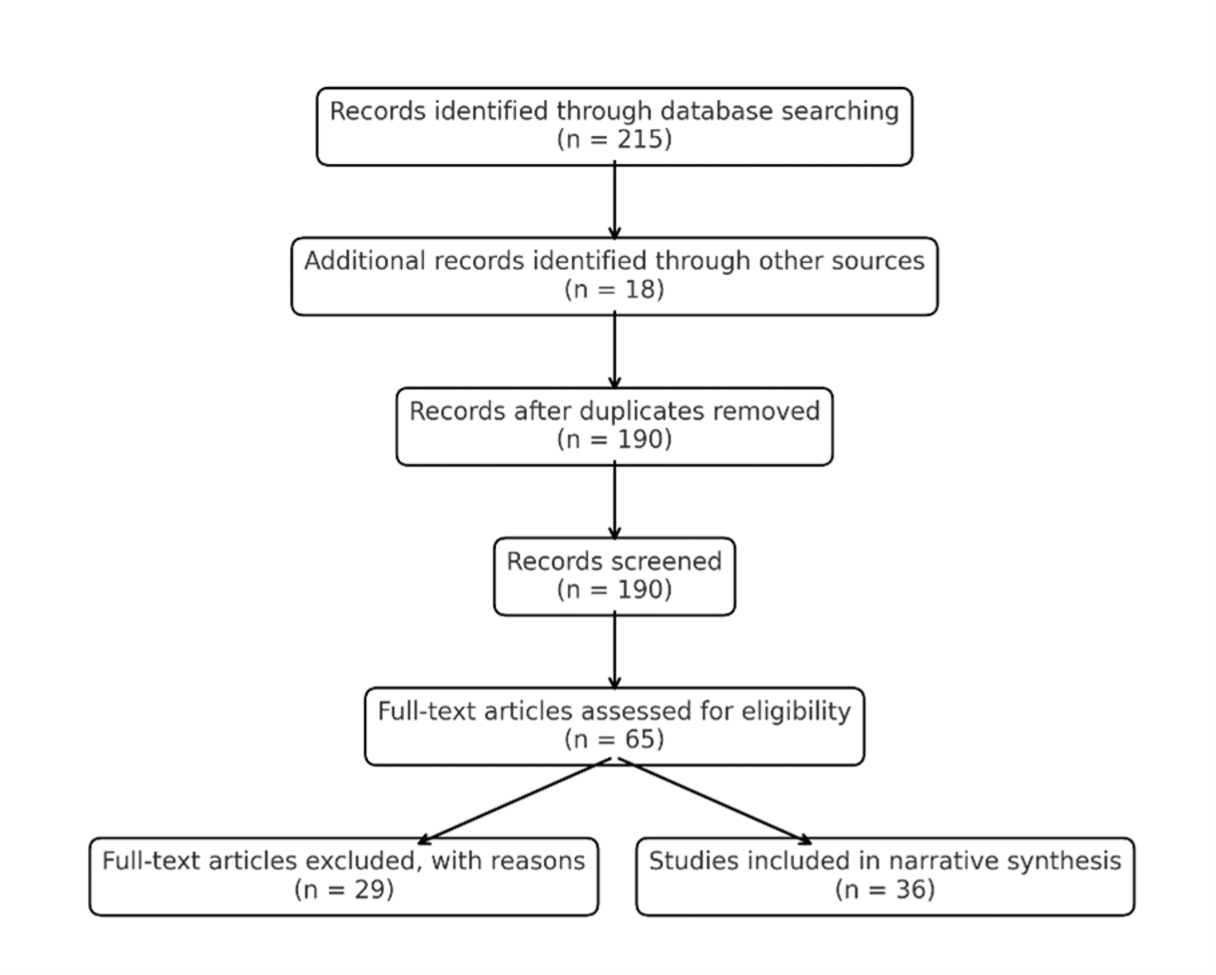
Literature search flow diagram

A structured literature search was conducted using systematic review databases and inclusion/exclusion criteria, but without formal risk-of-bias assessment or meta-analysis. The decision to adopt a narrative approach was intentional, allowing for the inclusion of recent feasibility, validation, and pilot studies that were heterogeneous in design and outcomes, which would not fit within a strict systematic framework. The initial database search yielded 215 records, with an additional 18 records identified through manual reference screening. After removing duplicates, 190 articles remained for title and abstract screening. Of these, 65 full-text articles were assessed for eligibility, and 29 were excluded for not meeting the inclusion criteria. Ultimately, 36 studies were included in this narrative synthesis. Figure 1 demonstrates the manual selection process of the literature.

Overview of devices to monitor glaucoma

The diagnosis of glaucoma requires a thorough investigation and ophthalmic examination: first testing visual acuity and then measuring IOP, followed by central corneal thickness (CCT), inspecting the angle with gonioscopy, examining the retina for structural and functional changes with optical coherence tomography (OCT) imaging, and, finally, VF testing [14].

Regular follow-up after diagnosing and initiating treatment is essential to detect disease progression and assess the response to treatment. This, however, is an added burden on health care costs.

Visual Acuity

Smartphone applications are a great, accessible way to reach patients and can be easily used without training. Samanta et al. investigated literature on smartphone-based visual acuity applications. The Peek Acuity app, which follows the standard Early Treatment Diabetic Retinopathy Study (ETDRS) chart design with a 5 × 5 grid optotype letter “E” displayed in one of four orientations, was excellent compared to clinic-based VA measurements. The mean difference between the smartphone acuities and Snellen/ETDRS acuities was 0.07 and 0.08 LogMAR, respectively [15]. Another study suggested that the Peek Acuity app is as accurate as the standard logMAR chart [16].

Another application is Odysight, which is used for near-VA testing. A study that included 88 participants in France showed excellent agreement with ETDRS but underestimated VA by 1.53 letters. The Vision at Home (V@home) application could be a reliable and accurate measure of both near and distance VA [17]. Finally, Snellen visual acuity applications were found to have considerable variability in accuracy and were the least reliable [18].

Optic Nerve Head Imaging

The purpose of these home-based smartphone imaging devices is to detect structural changes to the Optic disc, such as PPA, cup-to-disc ratio, notching, and disc hemorrhages.

First is the D-eye Adaptor, a small gadget mounted on a smartphone camera that allows the mobile camera to capture the fundus image [19]. A comparison study was done over 110 eyes with an undilated slit lamp in the clinic. The correlation between the smartphone and slit-lamp was 0.63; imaging was possible in 97% of eyes. A follow-up study using the same device suggested that using pupillary dilation improved comparability to the slit lamp [20].

Although smartphone-based optic nerve head (ONH) devices are promising for non-contact remote assessment, the dilation requirements and training needed are barriers to proper home access [14].

Tonometry (Intraocular Pressure Monitoring)

Since IOP is the only modifiable risk factor for glaucoma, obtaining accurate recordings at multiple times during the day is crucial, as IOP can fluctuate. Relying on a single clinic measurement may therefore be misleading.

One of the home-based tonometry devices is the iCare HOME. It is a handheld device that measures IOP for the user. It was approved by the FDA in March 2017. It can be used to capture early changes and enable earlier interventions. The device showed promising results compared to clinic-based devices, though some patients might have difficulty using it [21].

Overview of home-based visual field monitoring devices

Home-based VF testing is available with new devices or software for smartphones or tablets. Both methods, when integrated with artificial intelligence, compare well with clinic-based SAP [22]. Tablet or iPad-based systems are used with multiple devices such as the Melbourne Rapid Fields (MRF), VF-Home, and Eyecatcher [22].

MRF are software applications that can be used on a home tablet device, laptops, or desktop PC. This software uses ground-breaking technologies to perform VF test (perimetry), visual acuity, and other tests. It can be used in a clinic-based setting or at home. It then generates standardized charts and graphs for the ophthalmologist to assess glaucoma VF progression and formulate a treatment plan.

Eyecatcher is very similar to MRF, making an ordinary tablet equipped with eye and head tracking technologies a powerful portable testing device. The operation is simple; the patient sits in front of the tablet and is simply asked to look at anything that they see appears. Unlike SAP, there is no button response or central fixation. The software will track eye movements and analyze them to determine whether the user has seen the stimulus.

The devices showcase the convenience of being portable and use the front-facing camera for analysis of the VF, with ongoing machine learning for quality control [23]. Some devices, such as the Eyecatcher, integrate artificial intelligence and machine learning for anomaly detection, which then flags unreliable results to ensure maintenance of testing quality [24]. Others, such as the MRF, use voice commands and radial test pattern for improved accuracy, while the VF-home has the advantage of unsupervised home use for patient convenience [23]. Some suggest its use in clinics' waiting areas and screening patients.

Virtual reality headset platforms include the Vivid Vision Perimetry (VVP), Olleyes VisuALL, and multiple other devices reviewed by Hekmatjah et al., including Oculus Quest, VirtualEye, AVA, Radius, and VF [25]. They have the advantage of creating an immersive controlled environment for testing of the VF, with some incorporating eye tracking and proprietary threshold algorithms [26]. More recent platforms, such as the Virtual 7, offer better fixation monitoring and standardization of testing, which make them more advantageous compared to older platforms [25].

Evidence of clinical utility

Melbourne Rapid Fields

Several studies have compared MRF to the Humphrey Visual Field (HVF). In a 12-month study [27], glaucoma patients using at-home MRF showed good conformity results with in-clinic HVF. The reliability of home examination was, however, significantly lower than clinic tests (MRF: 65% vs. HVF: 85%). However, MRF successfully detected disease progression in two eyes, which were later confirmed by in-clinic assessments [22]. More recently, an extensive multi-center study compared MRF with HVF in 232 participants and found that MRF showed a high level of concordance with Humphrey Field Analyzer (HFA) without significant difference in test time, reliability indices, and fixation losses [24]. MRF seems to be a viable alternative test accessible at a lower cost in settings where HFA is inaccessible.

Several studies have investigated the difference between home-based VF testing and SAP, in which many reported the correlation coefficients for global indices (MD and pattern standard deviation) to range from 0.68 to 0.94 [28]. The area under the receiver operating characteristic curve (AUC) values for the detection of glaucoma were similar between the two testing methods, where one study found the AUC of MRF to be 0.84-0.89 compared to the Humphrey Field Analyzer AUC of 0.85 [29]. This shows promising overall results of home-based devices compared to SAP. However, one pronounced difference was the point-wise sensitivity of VF defects, especially with tablet-based MRF, which overestimated the nasal field and underestimated the temporal VF when compared to the Humphrey Field Analyzer [22].

Eyecatcher

The Eyecatcher tablet has also shown strong diagnostic potential in a small pilot study of 69 patients using Eyecatcher in glaucoma clinic waiting area [30]. There was a strong link between Eyecatcher’s mean hit rate and SAP MD. Crucially, no patients with substantial field loss were classified as visually normal. The sensitivity for identifying visually normal referrals was 73%, and the specificity for detecting eyes with moderate or advanced vision loss was 100%. Test-retest reliability was lower than HVF, yet it showed no learning or fatigue effects.

Vivid Vision Perimetry

Virtual Reality Perimetry has similarly performed well against in-clinic testing. In a pediatric feasibility study, Vivid Vision Perimetry (VVP) was administered to children between 7 and 18 years of age with best-corrected visual acuity of 20/80 or better due to various visual conditions including glaucoma. There was a moderate correlation between HVF mean sensitivity and VVP's mean “fraction seen” score (score 0.48), and 70% of participants favored the VVP [31]. The reliability of the VVP was not significantly different from that of HVF, and this has to do with the shortcomings of HVF, especially with fixation losses in the pediatric population.

A study on 41 eyes with glaucoma was conducted to determine the test-retest variability of the VVP Virtual Reality Perimetry device at home. A single remote training session was done for each subject on how to use the device, after which they were asked to do 10 tests alone over 14 days. The mean age group of participants was 62 years, and results have shown a low test-retest variability of VVP, and a high correlation between VVP and the standard in-clinic perimetry device HFA (Spearman’s correlation 0.87, 95% confidence interval, 0.66-0.98) in moderate-severe glaucoma cases but not in more mild cases. Most importantly, 95% of subjects could complete all 10 test sessions with a single remote training session, deeming VVP very easy to use [32]. In another small pilot on 42 eyes using remotely administered virtual reality VF devices compared to HVF, although it showed high rates of fixation losses and variability, users considered virtual reality devices comfortable and easy to use [33].

Patient adherence and engagement

Home monitoring devices for glaucoma have shown promising results in terms of usability. Most patients found the iCare home tonometer (HT) and VF devices easy to use. A satisfaction survey for a small cohort of trained individuals demonstrated that 73.7% of subjects reported that the HT was easy to use compared to 100% of the VF device users [34]. This suggests a relatively shallow learning curve for these devices. Additionally, patient satisfaction with home monitoring devices appears to be high, as all patients (100%) found the HT helpful device compared to 94.4% of the VF device users [34].

Studies have shown that patient adherence to home monitoring regimens can be pretty good. In one study, 88% of participants completed at least one home examination, and 69% completed all six requested examinations over several weeks [35]. Another study reported an impressive 98% adherence rate for monthly tests over six months [24].

Advantages of home-based monitoring

Disease Progression

Home-based VF monitoring for glaucoma delivers multiple benefits across several domains. Firstly, it increases the testing frequency and shortens the interval between tests, thereby aiding in early detection of disease progression. Multiple studies have shown that high-frequency home testing decreases variability in measurement, identifies vision changes earlier, and supports faster clinical intervention to prevent further disease progression compared to standard annual clinic visits [30]. This is particularly important to prevent the risks of delayed detection of disease progression. In a 12-month study, the MRF detected glaucoma progression approximately 10 weeks earlier than routine follow-up schedules with glaucoma specialists [27].

Patient Experience

At-home VF testing is also more convenient and accessible to all patients, especially with standard handheld devices such as tablets, PCs, and VR headsets. This also removes the need to travel to the clinic, saving time and reducing patients' stress and accessibility challenges [22,30]. The systems used are very comprehensive and user-friendly, improving patient adherence and providing better personalized patient care plans based on specific risk profiles of each patient [30]. The Eyecatcher software incorporates head-tracking technology, thereby eliminating the need for physical head restraints. Furthermore, it does not require manual button responses or central fixation, which enhances its ease of use and portability.

Healthcare System

The shift of VF monitoring from the clinic to home reduces clinic burden by decreasing appointments and waiting time, alleviating pressure on healthcare staff, and enabling resources to be focused on higher-risk patients who need it. This can eventually lead to more efficient clinic operation. Table 1 summarizes the findings, sample size, and limitations of key studies in this review. 

**Table 1:** Findings, sample size, and limitations of key studies. AUROC, area under the receiver operating characteristic curve; FL, fixation losses; FN, false negative; FP, false positive; HVF, Humphrey Visual Field; MAE, measurement error; MD, mean deviation; MRFh, Melbourne Rapid Fields; SAP, standard automated perimetry; VF, visual field; VVP, Vivid Vision Perimetry

Study Name	Sample Sizes	Outcomes (Key Findings)	Limitations (Primary Challenges)
Using an open-source tablet perimeter (Eyecatcher) as a rapid triage measure for glaucoma clinic waiting areas [30]	77 adults (including 11 new referrals)	Rapid: faster than SAP (median 2.5 min vs 3.5 min for SITA Fast). High specificity: 100% correctly identified eyes with moderate-to-advanced loss (MD < −6 dB). Good concordance: strong association with SAP MD (r=0.78). High usability: rated easier and less tiring than SAP.	Repeatability: less repeatable than conventional SAP (CoR95 for mean hit rate was 0.19). Technical failure: 9% could not complete due to eye-tracking hardware failure (often linked to recent surgery/interventions). VF coverage: unable to test central vision. Limited sensitivity (68%) for identifying false-positive referrals.
Test reliability and compliance to a twelve-month visual field telemedicine study in glaucoma patients [27]	47 participants enrolled; 20 participants analyzed (met criteria for progression analysis)	Good compliance: 75% adherence to weekly testing (97% adherence monthly). Early detection: confirmed progression in two patients 10 weeks earlier than scheduled clinical review. Strong concordance: high correlation between MRFh and HFA MDs (r=0.90 after 10 tests). Variability decreased significantly after 10 tests (learning phase identified).	Low retention: only 32% (15/47) remained active at 12 months. Moderate reliability: test reliability at home was 65% versus 85% for HFA in-clinic. 25% of subjects showed a learning effect resulting in artifactual improvement. Conducted during COVID-19, potentially boosting compliance rates.
Glaucoma home monitoring using online circular contrast perimetry over 6 months: performance and patient attitudes in the developing world setting [36]	20 adults (volunteers with established glaucoma).	High adherence: 98.3% of monthly tests completed successfully. Excellent accuracy: very good correlation between home (Eyecatcher) and clinic (HFA) VFs (r=0.94). Reduced error: adding home data to 2 standard SAP tests reduced MAE by more than 50% in 90% of eyes. Anomalous tests (9%) identifiable using machine learning/camera data.	Small pilot sample limits generalizability. Assessed only paracentral vision (limited field of view). Test implementation was described as crude (rudimentary ZEST algorithm). One participant discontinued due to dizziness/vertigo symptoms.
Home-based visual field test for glaucoma screening comparison with Humphrey perimeter [28]	10 patients (20 eyes, 1,040 test points analyzed).	Screening focus: supra-threshold algorithm intended only for screening, not monitoring progression. Good diagnostic ability: AUROC coefficients ranged from 0.762 to 0.837. Fast: test duration is 2–3 minutes per eye. Standardized: uses webcam as a "virtual photometer" to standardize dark room conditions and includes reliability validation (FL/FP/FN < 25%).	Very small sample size (10 patients). Comparison discrepancy: test used 24°/52 points, while HFA used 30°/76 points. Digital divide: older people often lack computer literacy, requiring support.
Pilot study comparing a new virtual reality-based visual field test to standard perimetry in children [31]	23 participants (37 eyes); average age 12.9 years.	High feasibility/acceptance: all participants successfully completed VVP. 100% preferred VVP over HVF. Similar test duration (approx. 3.9 minutes). Uses multifixation strategy, beneficial for children with fixation difficulties.	Lacks built-in reliability: VVP currently lacks definitive internal reliability metrics. Weak correlation: ONLY moderate correlation (R=0.48) observed when unreliable HVF tests were excluded; correlation was insignificant otherwise. Both VVP and HVF showed high unreliability rates (approx. 35–38% unreliable). VVP uses supra-threshold stimuli, making direct comparison to threshold HVF difficult.

Barriers to adoption

Despite growing interest in home-based VF monitoring devices for managing glaucoma, several barriers prevent their widespread use. Technical challenges are prevalent. One study reported that home perimeters show a mean test-retest variability of more than 3 dB, which raises some concerns regarding accuracy and reliability [24].

Patient-related factors also limit implementation. One challenge reported is adherence over time. In a longitudinal home-monitoring study, adherence rates were reduced to 75% and dropout was significant, with only 32% of the participants remaining until the trial's end. This, in turn, compromises the reliability of progression tracking. Factors that played a role in the low adherence rates include time management issues, lack of motivation, and digital illiteracy [27]. Another study cited a significant drop out at three- and six-month intervals due to similar factors [36].

Healthcare system barriers further reduce their use. Few studies reported that unclear reimbursement policies or EHR integration presented challenges. However, the logistical and infrastructural barriers, including access to technology and data transmission privacy, presented greater challenges [24].

Finally, clinician skepticism and inertia create notable obstacles. Qualitative findings showed participant and clinician distrust in test reliability and suggested solutions such as test repetition and confidence ratings. This implies being careful when relying on home-based testing data [24].

To address these barriers, it is best to start using training programs for patients to familiarize them with the system. Other strategies include standardized calibration protocols, clear reimbursement frameworks, secure data transmission systems, and validation studies of home-based VF monitoring devices, enhancing glaucoma management and decreasing the burden on in-clinic services. A summary of the three main VF monitoring software and devices is displayed in Table 2.

**Table 2:** Comparison of digital visual field assessment devices for glaucoma monitoring.

Device name	Melbourne Rapid Fields [37]	Eyecatcher [38]	Vivid Vision Perimetry [39]
Platform	Tablet or PC devices	Tablet or PC devices	Virtual reality headset
Key features	Browser-based access AI integrated Customizable protocols Multilingual support	Unsupervised use Eye tracking AI integrated Multilingual support	Immersive environment Eye tracking Favoured by patients
Validation status	High level of concordance with SAP	Strong diagnostic potential	High correlation between VVP and HFA (Spearman correlation 0.87)
References	Prea et al. 2022 [27]	Jones et al. 2021 [24]	Chia et al. 2024 [32]

Future directions

The first direction toward the future of home-based monitoring devices is conducting large-scale studies to assess and improve the sensitivity and specificity of certain devices, forming a standardized protocol for testing, interpretation of data, follow-up, and raising threshold for medical attention.

We suggest investing in low-cost, open-source VF applications that can be easily installed on tablet devices by users, enabling wide deployment even in low-income regions. In addition, integrating home-based VF tools into existing teleophthalmology networks for remote supervision and referral triage. This enables a hybrid model, ensuring testing with professional supervision without the need for frequent facility visits.

To encounter digital literacy and psychological acceptance by physicians and patients, a helpful yet simple training module, including tutorial videos targeting each physician and user separately, is crucial. More advanced training can be provided to community health workers and optometry assistants.

From a technological point of view, there is a need to make validated, user-friendly VA apps with easy-to-use instructions, as well as improving the ONH imaging devices, making it cost-effective, and reducing the need for dilation. Finally, the goal is to validate handheld OCT and tablet-based perimetry devices.

Increasing accessibility means a bigger set of data that requires a telemedical platform to store the data privately and be powered with AI tools to enhance filtering and triaging for healthcare providers’ concerns.

Public-private subsidy models and AI-assisted triage can further enhance cost-effectiveness and scalability, making home-based VF monitoring feasible across both developed and developing health systems.

## Conclusions

Glaucoma is a progressive and highly prevalent condition with a significant risk of irreversible vision loss if not detected early. VF testing remains central to monitoring disease progression. The expansion of telemedicine after the COVID-19 pandemic has accelerated the development of home-based monitoring devices, which show promise for earlier detection, improved accessibility, and reduced clinic burden. Despite challenges with reliability, adherence, and integration into existing systems, these technologies have strong potential to transform future glaucoma care.
